# Dance/movement therapy in psychotic disorders and eating disorders: a systematic review

**DOI:** 10.3389/fpsyt.2026.1802394

**Published:** 2026-04-23

**Authors:** Francesco Monaco, Annarita Vignapiano, Stefania Landi, Ernesta Panarello, Raffaele Malvone, Ilaria Pullano, Germano Fiore, Anna Maria Iazzolino, Luca Steardo, Gennaro Sosto, Giulio Corrivetti

**Affiliations:** 1 Department of Mental Health, ASL Salerno, Salerno, Italy; 2 European Biomedical Research Institute of Salerno (EBRIS), Salerno, Italy; 3 Psychiatric Unit, Department of Health Sciences, University Magna Graecia of Catanzaro, Catanzaro, Italy; 4 General Management, ASL Salerno, Salerno, Italy

**Keywords:** dance movement, eating disorders, psychotic disorder, rehabilitation, therapy

## Abstract

**Background:**

Dance/Movement Therapy (DMT) is a body-oriented form of creative arts therapy that uses movement and embodied awareness to support emotional regulation and psychosocial functioning. Interest in embodied and non-verbal interventions has increased in psychiatry, particularly for disorders characterized by disturbances in bodily experience and affect regulation, such as psychotic disorders and eating disorders (EDs). However, evidence on DMT remains fragmented and is often embedded within broader research on creative arts therapies.

**Objectives:**

This systematic review aimed to synthesize and critically evaluate the available evidence on DMT as an adjunctive intervention in psychiatric populations, with particular focus on psychotic disorders and EDs.

**Methods:**

A systematic search of PubMed/MEDLINE was conducted for studies published in English between January 2016 and December 2025. Eligible studies included randomized controlled trials, non-randomized or quasi-experimental studies, observational designs, and qualitative or mixed methods investigations involving adolescents or adults with psychotic disorders or EDs receiving DMT-based interventions. Risk of bias was assessed using RoB 2 and ROBINS-I tools. Due to methodological heterogeneity, findings were synthesized narratively.

**Results:**

Twelve studies met inclusion criteria. Across diagnostic groups, DMT was most consistently associated with improvements in embodied and psychosocial domains, including bodily awareness, emotional expression, and social functioning. In psychotic disorders, DMT was linked to improvements in negative symptoms, social engagement, and physical functioning when integrated into treatment as usual. Evidence in ED populations was more limited but suggested potential benefits for body image, emotional regulation, alexithymia, and subjective well-being. Qualitative findings highlighted increased emotional awareness, sense of agency, and relational attunement. Substantial heterogeneity in study design, intervention characteristics, and outcome measures limited causal inference.

**Conclusions:**

Current evidence suggests that DMT may represent a promising adjunctive intervention in psychiatric care, particularly for addressing embodied and relational dimensions of psychopathology. However, conclusions regarding efficacy remain tentative due to methodological limitations and variable study quality. Future research should prioritize well-designed controlled trials, standardized reporting of DMT protocols, and outcome measures sensitive to embodied change, with greater attention to underrepresented populations such as individuals with EDs.

**Systematic review registration:**

https://www.crd.york.ac.uk/prospero/, identifier CRD420261279779.

## Introduction

Dance/Movement Therapy (DMT) is a form of creative arts therapy that employs bodily movement, rhythm, and relational attunement as core therapeutic tools to support emotional regulation, self-awareness, and interpersonal engagement (1). Grounded in embodied, phenomenological, and developmental frameworks, DMT conceptualizes psychopathology as involving disturbances not only in cognition and affect, but also in bodily experience, motor expression, and implicit relational processes (2, 3). From this perspective, movement is understood not merely as a behavioral output, but as a primary medium through which emotional, cognitive, and relational processes are organized and expressed (2–5). In recent years, psychiatry has increasingly acknowledged the limitations of exclusively pharmacological and verbally mediated psychotherapeutic approaches, particularly in severe and complex mental disorders, highlighting the need to address embodied, interoceptive, and relational dimensions of psychopathology (6–8). A growing body of research indicates that many psychiatric conditions are characterized by alterations in embodied self-experience, impaired interoceptive awareness, psychomotor disturbances, and difficulties in affect regulation and social attunement—domains that are often insufficiently addressed by standard treatments alone (6–9). This recognition has contributed to renewed clinical and scientific interest in body-oriented and non-verbal interventions as adjunctive components within multidisciplinary mental health care. Within this broader framework of embodied and expressive interventions, DMT has been proposed as a particularly relevant therapeutic modality for psychiatric populations in which verbal communication may be limited or insufficient to access core experiential dimensions of distress (5). Through movement, posture, spatial exploration, mirroring, and shared rhythmic activity, DMT may facilitate access to pre-reflective and sensorimotor levels of experience, supporting processes of emotion regulation, bodily integration, and relational engagement (3, 4). These mechanisms are especially pertinent in disorders marked by negative symptoms, psychomotor alterations, body image disturbances, and social withdrawal. Empirical research on DMT has examined its application across a range of psychiatric conditions, with a substantial proportion of studies focusing on schizophrenia spectrum and other psychotic disorders (3, 5, 10). Quantitative studies suggest that, when integrated into treatment as usual, DMT may contribute to improvements in negative symptoms, motivation, psychosocial functioning, and aspects of physical health (11–14). Although effect sizes and outcome domains vary across studies, these findings are clinically relevant given the limited responsiveness of negative symptoms to pharmacological treatment alone. In parallel, qualitative and mixed-methods investigations have consistently highlighted experiential benefits of DMT in psychotic disorders, including enhanced emotional expression, stress relief, sense of agency, and improved interpersonal attunement (15–17). Beyond psychotic disorders, DMT has also been explored in the treatment of eating disorders (EDs), which are characterized by profound disturbances in body image, interoceptive awareness, emotional regulation, and embodied self-experience. Individuals with EDs frequently report estrangement from bodily sensations, heightened somatic tension, and rigid or avoidant movement patterns, suggesting that interventions directly targeting bodily experience may be particularly relevant (6, 18, 19). Emerging evidence indicates that DMT may support improvements in body awareness, emotional expression, alexithymia, and subjective well-being in individuals with anorexia nervosa and related disorders, potentially complementing cognitively and nutritionally oriented treatments (15, 20, 21). However, the evidence base in ED populations remains limited and largely exploratory. Importantly, evidence from other creative and expressive arts therapies—such as art therapy and mixed expressive interventions—has also demonstrated beneficial effects on emotional expression, psychosocial functioning, and experiential dimensions in psychiatric populations (22–24). While these findings provide important contextual support for non-verbal and embodied therapeutic approaches, they should not be conflated with evidence specific to DMT, given differences in theoretical foundations, therapeutic mechanisms, and modes of engagement. Distinguishing DMT-specific effects from those of the broader field of creative expressive arts therapies is therefore essential for both clinical interpretation and research synthesis. Despite increasing clinical interest and theoretical support, the empirical evidence base for DMT remains heterogeneous. Previous reviews have identified substantial methodological limitations, including small sample sizes, variability in intervention protocols, heterogeneity of outcome measures, and a predominance of non-randomized designs (1, 3, 13). Moreover, many existing syntheses have examined creative arts therapies collectively rather than focusing specifically on DMT as a distinct modality, thereby limiting the specificity of conclusions regarding its unique mechanisms and clinical effects. Several studies published in recent years—including randomized trials, mixed-methods investigations, and mechanism-oriented contributions—have not yet been fully integrated into comprehensive systematic syntheses (14, 20, 21, 25). While this review primarily focuses on DMT, evidence from other creative and expressive arts therapies is considered to contextualize DMT findings within the broader field of embodied and non-verbal psychiatric interventions. Against this background, the present systematic review aims to synthesize and critically evaluate the current evidence on the effectiveness, clinical outcome. Psychotic disorders and EDs were selected as the primary focus of the present review because both conditions are characterized by profound disturbances in embodied self-experience, interoceptive processing, and the relationship between bodily sensations and emotional regulation. In schizophrenia spectrum disorders, disruptions of minimal selfhood, psychomotor functioning, and interpersonal attunement are well documented, often accompanied by negative symptoms and altered bodily awareness (2, 7, 26). Similarly, EDs are strongly associated with disturbances in body image, interoceptive awareness, and embodied self-experience, which play a central role in their psychopathology (6, 18). Because DMT directly targets embodied processes through movement, bodily awareness, and relational attunement, these two diagnostic groups represent clinically relevant populations in which the mechanisms of DMT may be particularly meaningful. Examining these conditions within a single review also allows exploration of potential transdiagnostic mechanisms related to embodiment and affect regulation that may extend across distinct psychiatric disorders and therapeutic mechanisms of DMT in psychiatric populations. By focusing specifically on DMT, this review seeks to clarify its role as an adjunctive intervention in mental health care, examine patterns of response across diagnostic groups and treatment settings, and identify methodological priorities for future research.

## Methods

### Search strategy

A systematic literature search was conducted in the electronic database PubMed/MEDLINE to identify relevant studies examining creative and expressive arts therapies in psychiatric populations, with a specific focus on DMT. The review protocol was registered on PROSPERO (CRD420261279779). The search covered studies published between January 2016 and December 2025 in order to capture the most recent decade of empirical research while avoiding redundancy with earlier systematic reviews in the field. PubMed/MEDLINE was selected because it provides comprehensive coverage of peer-reviewed biomedical and psychiatric literature and is widely regarded as a reference database for clinical and mental health research. To further reduce the risk of missing relevant publications, the reference lists of all included studies and relevant review articles were manually screened and it was restricted to articles published in English. Only studies involving human participants were considered. The search strategy combined controlled vocabulary terms (e.g., MeSH terms) and free-text keywords related to DMT and creative/expressive arts therapies, in conjunction with terms referring to psychiatric disorders, with particular attention to psychotic disorders and EDs. This broader strategy was adopted to ensure sensitivity of the search and to capture studies in which DMT was examined within the wider field of creative or expressive arts therapies. Filters were applied to exclude systematic, narrative, or scoping reviews, meta-analyses, case reports, conference abstracts, editorials, book chapters, and other non–peer-reviewed material.

The following search string was used, with adaptations according to the syntax of each database:

((“dance movement therapy” OR “dance/movement therapy” OR “dance therapy” OR “creative arts therapy” OR “expressive arts”) AND (“psychotic disorders”[MeSH Terms] OR psychosis OR schizophrenia OR “eating disorders”[MeSH Terms])) AND (treatment OR therapyORrehabilitation).

AND (“2016/01/01”[Date – Publication]: “2025/12/31”[Date – Publication]).

In most indexed studies, the term “dance movement therapist” appears in relation to the professional delivering the intervention rather than the intervention itself; therefore, the use of therapy-related keywords was considered sufficient to retrieve relevant studies. The MeSH term “eating disorders” was used to capture the spectrum of diagnostic entities within this category, including anorexia nervosa, bulimia nervosa, and binge eating disorder, which are indexed under this broader heading in PubMed.

The reference lists of all included articles and relevant reviews were manually screened to identify further eligible studies not retrieved through the electronic search. Key journals in the field of dance and movement psychotherapy, including Body, Movement and Dance in Psychotherapy, were also consulted to ensure that potentially relevant studies were not missed. Although the primary focus of the present review is DMT, a broader search strategy including creative and expressive arts therapies was intentionally adopted in order to contextualize DMTspecific findings within the wider field of embodied and non-verbal psychiatric interventions. Only studies explicitly reporting DMT as a core intervention were considered central to the analytic synthesis, while evidence from other expressive modalities was used for contextual and comparative purposes.

### Eligibility criteria

Study eligibility was defined a priori using a structured PICO framework, specifying the target population (psychosis and eating disorder populations), the intervention of interest (creative and expressive arts–based interventions), comparators (when applicable), and relevant clinical, embodied, and psychosocial outcomes, with dance/movement therapy defined as the primary modality of interest. Interventions encompassed a range of creative and expressive modalities, including dance/movement-based therapies, art-based approaches, and other structured expressive interventions. DMT was considered a primary modality of interest and is discussed in greater detail throughout the review.

### Population

Studies were eligible if they included adolescent or adult participants (≥12 years) diagnosed with a psychiatric disorder or eating disorder according to DSM-5-TR, ICD-10. Eligible diagnostic groups included:

- Psychotic disorders, such as schizophrenia spectrum disorders, schizoaffective disorder, and first-episode psychosis;
- Eating disorders, including anorexia nervosa, bulimia nervosa, binge-eating disorder, and other specified feeding or eating disorders.

Psychotic disorders and EDs were selected because they are characterized by profound disturbances in embodiment, identity, affect regulation, and interpersonal functioning, which are central targets of creative and expressive arts–based interventions, including DMT. Studies focusing exclusively on healthy, non-clinical populations were excluded. Psychotic disorders and EDs were specifically selected because both are characterized by prominent disturbances in embodied self-experience and affect regulation, domains that represent core theoretical targets of DMT.

### Intervention

The interventions of interest were creative and expressive arts therapies, defined as structured, therapist-led interventions using artistic or movement-based modalities within a therapeutic framework. Within this broader category, DMT was defined as a movement-based, embodied psychotherapeutic intervention explicitly grounded in DMT principles and delivered by trained professionals.

Studies examining general physical activity, recreational dance, yoga, or other movement-based practices not explicitly defined as DMT were excluded.

### Comparator

Studies were eligible regardless of the presence of a comparator group. When present, comparator conditions included treatment as usual (TAU), waitlist controls, or other active therapeutic interventions.

### Outcomes

Eligible studies were required to report at least one outcome within the following domains:

- Clinical outcomes, including symptom severity, diagnostic status, or psychopathological dimensions;
- Emotional and affective outcomes, such as emotion regulation, affective expression, stress, or alexithymia;
- Embodied or somatic outcomes, including body image, bodily awareness, interoceptive functioning, somatic tension, or motor functioning;
- Psychosocial and functional outcomes, such as quality of life, social functioning, or interpersonal engagement;
- Qualitative outcomes, including patient-reported experiences, perceived benefits, or therapeutic processes.

### Study design

The following study designs were considered eligible:

- Randomized controlled trials (RCTs);
- Non-randomized controlled studies;
- Quasi-experimental studies;
- Observational studies (cohort, case–control, cross-sectional);
- Pilot and feasibility studies with a clearly described methodology;
- Qualitative and mixed-methods studies.

### Exclusion criteria

Studies were excluded if they:

- Were review articles, editorials, commentaries, letters, or conference abstracts without full-text availability;
- Were single-case reports lacking a systematic methodology;
- Did not clearly describe the intervention as DMT;
- Involved non-clinical populations or animal models;
- Were not published in English.

### Study selection

**Figure 1:**
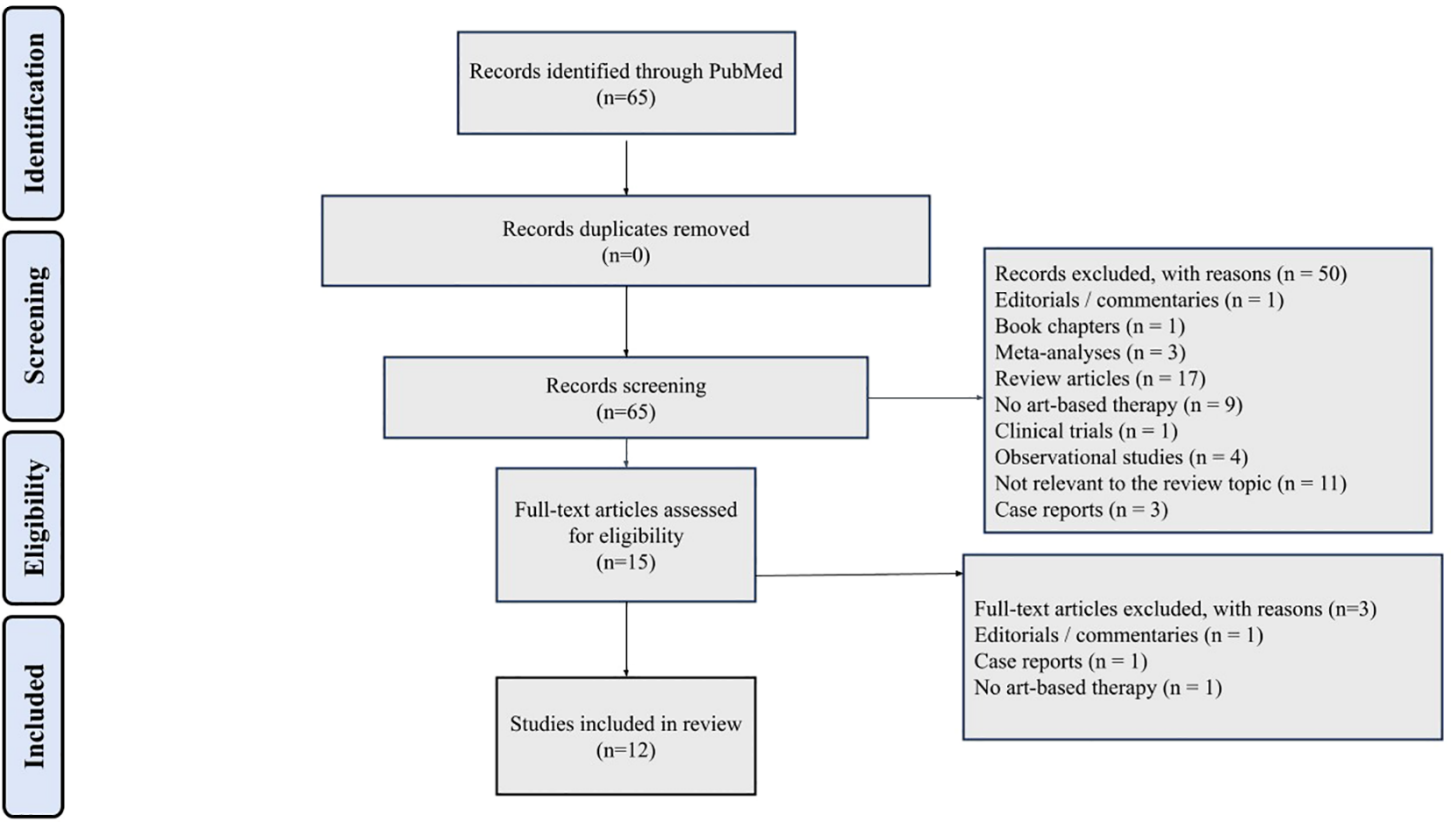
PRISMA flow diagram.

All records retrieved through the database searches were imported into a reference management system, and duplicate records were removed. Titles and abstracts were independently screened by two reviewers according to the predefined eligibility criteria. Full-text articles were subsequently assessed for inclusion when titles and abstracts suggested potential eligibility. Disagreements between reviewers were resolved through discussion and consensus; when necessary, a third reviewer was consulted. The study selection process followed PRISMA 2020 guidelines, and reasons for exclusion at the full-text stage were documented and reported in the PRISMA flow diagram (**Figure 1**).

### Data extraction

Data were independently extracted by two reviewers using a standardized and pilot-tested data extraction form. The following information was collected from each included study:

- General study characteristics (authors, year of publication, country, study design, and setting);
- Participant characteristics (sample size, age range, gender distribution, diagnostic criteria, and clinical population);
- Intervention characteristics (type of DMT, session frequency and duration, total intervention length, format, and therapist qualifications);
- Comparator details, when applicable;
- Outcome measures (instruments used, outcome domains assessed, and timing of assessments);
- Main findings (quantitative results, qualitative themes, and direction of effects);
- Follow-up data, when available.

### Quality assessment

Risk of bias was assessed independently by two reviewers according to study design.

Randomized controlled trials were evaluated using the Cochrane Risk of Bias Tool version 2 (RoB 2), while non-randomized intervention studies were assessed using the Risk Of Bias In Nonrandomized Studies of Interventions (ROBINS-I). Any discrepancies were resolved through discussion or consultation with a third reviewer.

### Data synthesis

Given the substantial heterogeneity in study designs, populations, intervention characteristics, and outcome measures, the findings were synthesized narratively. Results were organized according to diagnostic group (psychotic disorders vs. EDs) and intervention characteristics, with particular attention to DMT approaches. A quantitative meta-analysis was not considered appropriate due to the variability across studies. Consequently, no pooled effect sizes were calculated and results were interpreted descriptively.

## Results

### Study selection

The systematic database search identified a total of 65 records. After removal of duplicates, titles and abstracts were screened for eligibility. Following this initial screening, 50 records were excluded, primarily because they did not investigate DMT, involved non-clinical populations, or failed to meet the predefined inclusion criteria. Full-text assessment was conducted for 15 articles. Of these, 3 studies were excluded due to the intervention not being explicitly defined as DMT, insufficient methodological detail, inclusion of non-psychiatric populations, or ineligible study design. Consequently, 12 studies met all eligibility criteria and were included in the qualitative synthesis. The study selection process is summarized in the PRISMA 2020 flow diagram (**Figure 1**).

### Study characteristics

The included studies encompassed a range of methodological designs, including randomized controlled trials, non-randomized controlled studies, quasi-experimental designs, observational studies, and qualitative or mixed-methods investigations. This diversity reflects the exploratory and evolving nature of the evidence base on DMT in psychiatric populations. Sample sizes varied considerably across studies, ranging from small pilot samples to larger clinical cohorts. Most interventions were conducted in inpatient or residential psychiatric settings, although several studies also examined outpatient and community-based programs. It should be noted that, while the evidence base for DMT was more robust in psychotic disorders, studies addressing EDs were fewer and often embedded within broader creative or body-oriented therapeutic frameworks rather than exclusively DMT-specific interventions. A detailed overview of study designs, populations, intervention characteristics, and outcome domains is provided in **Table 1**.

**Table 1:** Overview of included studies.

Author	Year and country	Period	Study design	Study sample	Measures	Typeof intervention	Diagnosis	GRADE assessmentof articles	Outcome
Biondo & Gerber	2020, United States	Not reported (data collected over~4.5 months)	Qualitative observational study; secondary qualitative content analysis of therapist field notes froma Mixed methods feasibility study	N=32 inpatients with psychotic disorders (17 DMT intervention, 15 TAU); adults aged 18–62; mixed gender and ethnicity; sessions conducted in groups of 4–8 participants	BPRS (Brief Psychiatric Rating Scale)	Single-session Dance/MovementTherapy (DMT)	Schizophreniaand schizoaffective disorders (bipolar, depressive, mixed, unspecified types)	Low	Identification of consistent components of a single-session dance/movementtherapy protocol (warmup, thematic development, cooldown, verbal discussion). Findings suggest short-term symptom stabilization, improved mood, grounding, self-awareness, and peer connection duringacute inpatient hospitalization.
Bozzatello et al.	2018, Italy	January– December 2014(6month interventionand follow-up)	Prospective randomize d comparative study in a real-world outpatient rehabilitation setting	N = 54 adults with schizophreniaspectrum disorders (29 Befriending, 25 Group Art Therapy completers); mean age 46.8 ± 12.8 years; 55% male; long illness duration (mean18.7 years)	(CGIS )Clinical Global- Impression-Severity Scale; (CISS) Coping Inventory for Stressful Situations; (RSE) Rosenberg Self- Esteem Scale, (GAF), Global Assessmentof Functioningscale, (PSP), Personal and Social Performance Scale.	Group Art Therapy	Schizophrenia spectrum disorders (schizophrenia,schizoaffective disorder, delusional disorder, briefpsychotic disorder, other psychotic disorders)	Moderate	Both Befriending and Group Art Therapy led to significant within-group improvements in psychosocial functioning, self-esteem, and thought disturbance. Befriending was superior to Art Therapy in improving psychosocial functioning and emotion-oriented coping strategies, while no between-group differences were observed for core psychotic symptoms.
Bryl et al.	2020, Poland	Not reported (10-week intervention period	Mixed methods pilot study with randomized controlled trial and qualitative interviews	N = 31 outpatients with chronic schizophrenia (18 DMT, 13 TAU); adults aged 29–67 years; long illness duration; qualitative interviews completed by 15 DMT participants	PANSS; BNSS; WHO- DAS 2.0; SDS	Dance/Movement Therapy (DMT)	Schizophrenia (chronic, with prominent negative symptoms)	Low	Quantitative analyses showed no significant improvement in negative symptoms or psychosocial functioning in the DMT group compared with TAU. Qualitative findings suggested perceived benefits of DMT, including enhanced interpersonal connectivity, emotional support, self-integration, increased motivation, physical activation, and subjective improvement in management of negative symptoms.
Cho & Lee	2018, South Korea	November 2016– January 2017(6week intervention with 2week follow-up)	Quasi-experimental controlled before-and-after study with follow-up	N=35 inpatients with chronic schizophrenia(17 experimental, 18control); adults hospitalized ≥2 years; mixed gender	SANS; MAP-SR; Interpersonal Relationship Functioning AssessmentScale; Personal Sanitary ComplianceTable, Program Participation Table	Motivational Interviewing– BasedGroup Art Therapy	Schizophrenia (chronic)	Moderate	Motivational interviewing integrated with groupart therapy produced significantly greater improvements than control in negative symptoms, motivation and pleasure, interpersonal relationship functioning, personal hygiene compliance, and psychiatric ward program participation at post-testand follow-up.
Ciufalo et al.	2024, Italy	Not reported (12-week intervention period)	Doubleblinded controlled pilottrial with parallel groups	N = 24 patients with schizophrenia (12 Creative Puppet Therapy,12 pseudotreatment) and12 healthy controls; adults aged ~45 years; balanced gender distribution	CAPS; EEXI, Face Features of Puppets	Creative Puppet Therapy (CPT)	Schizophrenia (DSM-5)	Moderate	Creative Puppet Therapy (CPT) produced a large reduction in overall hallucination frequency compared with pseudotreatment, with pronounced decreasesin external auditory hallucinations and associated distress, suggestinga potential role in modulating perceptual and emotional aspectsof psychotic experience.
Cataldas et al.	2025, Turkey	December 2018– April 2019 (16-week intervention)	Quasi-experimental pre–post study with nonrandomizedcontrol group	N=14 Community dwelling adults with schizophrenia (7 art therapy, 7 control); aged 18–60; majority female;all receiving standard community mental health services	QLS; SFS	Intermodal Expressive Arts Therapy (GroupArt Therapy)	Schizophrenia(DSM-5; clinically stable, non-acute)	Low	Group art therapy was associated with improvements in overall quality of life and social functioning, particularlyin interpersonal relationships, role functioning, social participation, and independence. No significant changes were observed in the control group, andbetween group differences werenot statistically significant, likely due to limited sample size.
Kong et al.		2024, China	June 2019 –June 2020 (6–12 week intervention)	N=120 hospitalized male patients with chronic schizophrenia (60 DMT, 60 control); aged 30–60; long illness duration; stable pharmacological treatment	PANSS	DanceArt Therapy	Chronic schizophrenia	High	Compared with standardcare alone, dance art therapy significantly reduced positive, negative,and general psychopathological symptoms, improved executiveand global cognitive functioning, andledto greater reductionsin BMI at both 6 and 12 weeks. Effectswere consistently stronger in the intervention group across all outcome domains.
Qiu et al.	2017, China	July 2012 – February 2015(16week primary intervention; follow- up up to 12 months)	Randomize d, longitudinal, controlled trialin prison settings	N=120 prison inmates with schizophrenia randomized (final analyzed n = 105); adults 18–65; majority male;on stable antipsychotic treatment	S‐AG, state anger;TAG,trait anger; AC, anger control; SAX,state anxiety; TAX,trait anxiety; BDI, beck depression inventory; PANSS	GBTS Art Brut Therapy	Schizophrenia(DSM-5; various subtypes)	High	Theartbrut therapy program reduced emotional distressand negative symptoms, and improved behavioral regulation, social functioning, treatment adherence, and sleeppatterns compared with control. Effects emerged within 8–16weeks, werelargely maintained over follow-up, and were more pronounced for negativethan positive symptoms.
Romm et al.	2023, Norway	Not reported (12-week intervention period)	Qualitative exploratory study based on post-interventionsemi- structured interviews	N = 5 young adults (from 8 enrolled) receiving treatment for early psychosis; aged18–30; mixed inpatient and outpatient setting	Semistructured qualitative interviews; STC	Creative Writing Group	Early psychosis (first- episode psychosis; schizophrenia spectrum not further specified)	Low	Creative writingwas experienced as a meaningful recovery-oriented intervention, fostering connectedness, empowerment, self-expression, identity development beyond illness, and emotional awareness withina supportive and structured groupcontext, consistent with CHIME recovery dimensions ratherthan symptom reduction.
Sarandöl et al.	2024, Turkey	Not reported (17-week interventionwith follow-up up to 12 months)	Randomize d comparative study of two active psychosocial interventions	N=15 outpatients with schizophrenia (7 art therapy, 8 psychosocial skills training) and 12patient relatives; adultsaged 18–65; clinically stableon maintenance medication	PANSS, CDSS, SFS, HT, UOT, ZBI,BAI, BDI	GroupArt Therapy	Schizophrenia (DSM-5)	Moderate	Both art therapy and psychosocial skillstraining reduced negative symptoms and general psychopathology and improved socialand cognitive functioning over time. Art therapy showed broader improvements acrossspecific PANSS negative symptom domains, while PSSTeffects weremore limited.No significant between-group differences were observed, likely dueto small size. sample
Sprotte	2023, Germany	May– October 2016(6month intervention period)	Explorative longitudinal single case study (N-of-1 series) using computerized text and voice analysis during groupart therapy with therapist-guided picture reflection	N=7 inpatients withlong- term chronic schizophrenia; aged 46–62; mixed gender; judicially accommodated;high adherence across repeated sessions	Semistructured individual interviews	GroupArt Therapy with Therapist- Guided Picture Reflection (TGPR)	Chronic schizophrenia(ICD-10 F20.0, F20.5)	Low	Arttherapy with therapist-guided picture reflection was associated with increased linguistic presenceand reducedvocal markersof stressand anxiety. Individual trajectories were heterogeneous, withsome participants showing emotional activationand cathartic processes, suggesting computerized speech and voice measures as surrogate markers of process-related change.
Ursuliak et al.	2019, Canada	Not reported (13-week intervention with 3month follow-up)	Qualitative feasibility study with thematic analysis of post-interventionand follow-up interviews	N = 13 young adultswith earlyphase psychosis (from21 recruited); aged16–30; mixed gender; participants within 5 years of diagnosis; high engagement (62% retention, 80% attendance)	GAF, Semistructured individual interviews	Claymation GroupArt Therapy	Early phase psychosis (schizophrenia spectrum disorders, DSM-IV)	Low	Claymation group art therapy was feasible and highly engaging, with recovery-oriented benefits including stress relief,self- discovery, pride increative work, increased confidence and hope,social connectedness, andsustained engagement in meaningful activitiesat follow-up, aligningwith personal recovery domains rather than symptom reduction.

Summary of the main characteristics of the studies included in the systematic review. The table reports authors and year of publication, country of origin, study design, clinical population (psychotic disorders and/or eating disorders), sample size, characteristics of the DMT intervention (format, duration, and session frequency), comparator conditions when applicable, and primary outcome domains assessed. Included study designs comprise randomized controlled trials, non-randomized controlled studies, quasi-experimental designs, observational studies, and qualitative or mixed-methods investigations. EDs, eating disorders; DMT, Dance/Movement Therapy; TAU, treatment as usual.

### Characteristics of DMT interventions

DMT interventions varied substantially in terms of duration, frequency, and format. Most studies implemented group-based interventions, although individual formats were also reported. Session frequency ranged from once weekly to multiple sessions per week, and total intervention duration varied from single-session protocols to multi-week programs. Despite this variability, several core components were consistently described across interventions. These commonly included structured warm-up phases, guided movement exploration, relational or mirroring exercises, grounding techniques, and verbal reflection or integration phases; trained dance/movement therapists or clinicians with specialized training in DMT to deliver interventions. However, the level of detail provided regarding therapist qualifications and intervention manuals varied across studies.

A comprehensive overview of study characteristics, participant populations, intervention formats, and outcome domains is presented in **Table 1**.

### Outcomes in psychotic disorders

In studies involving individuals with psychotic disorders, DMT was most commonly associated with improvements across several outcome domains. These included reductions in negative symptoms, such as emotional blunting, social withdrawal, and reduced motivation, as well as improvements in psychosocial functioning, including greater interpersonal engagement, participation, and social interaction. In addition, several studies reported enhanced bodily awareness and physical functioning, reflected in improved movement fluency and somatic regulation. Quantitative studies frequently reported statistically significant pre–post improvements in symptom severity or functional outcomes when DMT was delivered as an adjunct to treatment as usual, although effect sizes varied across studies. Qualitative findings further emphasized experiential benefits, including increased emotional expression, enhanced sense of agency, and improved relational attunement following participation in DMT programs.

### Outcomes in eating disorders

In studies involving individuals with EDs, DMT was associated with improvements across several outcome domains. These domains were derived through thematic grouping of the outcome measures reported in the included studies, given the heterogeneity of assessment tools and outcome variables. Across studies, the most frequently reported improvements concerned body image and body awareness, emotional regulation and affective expression, and reductions in alexithymia and body-related distress. Movement-based therapeutic processes were frequently described as facilitating a more flexible and integrated relationship with the body, supporting patients’ capacity to tolerate bodily sensations and engage with emotional experiences. However, most studies involving ED populations were characterized by relatively small sample sizes and non-randomized designs, which limits the strength of causal inferences regarding the observed effects.

### Shared outcome domains across diagnostic groups

Across both diagnostic groups, several overlapping outcome domains emerged. In particular, improvements in embodied awareness, emotional expression, and psychosocial functioning were reported in studies involving both psychotic disorders and EDs. These shared outcomes suggest that DMT may act on transdiagnostic mechanisms related to embodiment, affect regulation, and interpersonal engagement, which are relevant across distinct psychiatric conditions.

### Qualitative and experiential outcomes

Across diagnostic groups, qualitative and mixed-methods studies consistently emphasized experiential and process-related benefits of DMT that were not always fully captured by standardized symptom measures. Commonly reported themes included increased emotional awareness, stress reduction, enhanced self-perception, and improved interpersonal connection. These findings underscore the importance of integrating qualitative and quantitative evidence when evaluating embodied therapeutic interventions.

### Risk of bias assessment

Risk of bias assessments are summarized in **Table 2** (RoB 2) for randomized controlled trials and **Table 3** (ROBINS-I) for non-randomized studies. Among randomized controlled trials, overall risk of bias ranged from low to some concerns, with the most frequent issues related to deviations from intended interventions and outcome measurement. Non-randomized studies frequently exhibited moderate to serious risk of bias, primarily due to confounding, participant selection, and lack of blinding.

**Table 2:** Evaluation of the risk of bias for randomized studies using the RoB 2.0 tool.

Reference	DMT intervention	Overall risk	Randomization	Intervention	Missing data	Outcome measurement	GRADE
20	CPT	Moderate	Low	Moderate	Low	Low	Moderate
14	DMT	Low	Low	Low	Low	Low	High
23	GBTS	Moderate	Low	Moderate	Moderate	Low	High

This table reports the risk of bias assessment for randomized controlled trials included in the systematic review, evaluated using the Cochrane Risk of Bias Tool version 2 (RoB 2). Bias was assessed across the following domains: bias arising from the randomization process, bias due to deviations from intended interventions, bias due to missing outcome data, bias in measurement of the outcome, and bias in selection of the reported result. Each domain and the overall risk of bias were rated as low risk, some concerns, or high risk, according to the RoB 2 guidance. CPT, Creative Puppet Therapy; DMT, Dance Art Therapy; GBTS, Art brut therapy.

**Table 3:** Evaluation of risk of bias in non-randomized studies using the ROBIN-I tool.

Study	Confounding	Selection of participants	Classification of intervention	Missing data	Measurement of outcomes	Overall risk
27	Serious	Moderate	Low	Low	Serious	Serious
11	Moderate	Moderate	Low	Moderate	Low	Moderate
13	Serious	Moderate	Low	Moderate	Moderate	Serious
16	Serious	Serious	Low	Moderate	Serious	Serious
21	Serious	Moderate	Low	Moderate	Moderate	Serious
25	Serious	Serious	Low	Moderate	Serious	Serious
15	Serious	Serious	Low	Moderate	Serious	Serious
28	Serious	Serious	Low	Serious	Moderate	Serious

This table summarizes the risk of bias assessment for non-randomized studies included in the systematic review, conducted using the Risk Of Bias In Non-randomized Studies of Interventions (ROBINS-I) tool. Bias was evaluated across the following domains: bias due to confounding, bias in selection of participants into the study, bias in classification of interventions, bias due to deviations from intended interventions, bias due to missing data, bias in measurement of outcomes, and bias in selection of the reported result. Overall risk of bias was categorized as low, moderate, serious, or critical in accordance with ROBINS-I criteria.

Overall, methodological quality varied substantially across studies, highlighting the exploratory nature of the current evidence base and the need for more rigorous controlled investigations.

#### Synthesis of findings

Overall, the available evidence suggests that creative and expressive arts therapies may be associated with improvements across multiple clinical, embodied, and psychosocial domains in psychiatric populations, particularly in relation to emotional regulation, bodily awareness, and interpersonal functioning. Within this heterogeneous body of literature, DMT interventions appeared to show particular promise for embodied and relational outcomes; however, substantial heterogeneity in study design, intervention characteristics, outcome measures, and methodological quality limits the ability to draw definitive conclusions regarding efficacy.

## Discussion

Overall, the findings of this review suggest that creative and expressive arts therapies may be associated with beneficial effects across embodied, emotional, and psychosocial domains in psychiatric populations. Within this broader field, DMT appears to be a particularly promising modality for outcomes related to bodily awareness, affective expression, and interpersonal functioning, although the strength of the evidence remains constrained by methodological heterogeneity across studies. The present findings are consistent with previous systematic reviews examining body-oriented and creative arts therapies in psychiatric populations. For example, Koch et al. (3) reported that dance and dance/movement therapy interventions are associated with improvements in psychological well-being and affect regulation across clinical and non-clinical populations. Similarly, Millman et al. (5) highlighted the potential role of movement-based interventions in supporting emotional processing and embodied awareness in mental health contexts. Reviews of art-based and body-oriented therapies in EDs have also emphasized the importance of addressing disturbances in bodily self-experience and interoception through experiential interventions (29). Taken together, these findings suggest that movement-based and other creative therapeutic modalities may complement verbally mediated treatments by targeting embodied dimensions of psychopathology. Importantly, despite examining psychotic disorders and EDs as distinct diagnostic categories, several overlapping outcome domains emerged across both groups. In particular, improvements in embodied awareness, emotional expression, and psychosocial functioning were consistently reported in studies involving both populations. These shared patterns suggest that DMT may act on transdiagnostic mechanisms related to embodiment, affect regulation, and interpersonal engagement. From this perspective, movement-based therapeutic processes may help address core disturbances in bodily self-experience and emotional processing that cut across different psychiatric conditions, rather than being specific to a single diagnostic category. Both psychotic disorders and EDs have been associated with disturbances in interoceptive processing, bodily self-experience, and affect regulation. By directly engaging sensorimotor processes, movement exploration, and relational attunement, DMT may provide therapeutic pathways that complement verbally mediated treatments, facilitating access to emotional and bodily experiences that are often difficult to articulate through conventional psychotherapeutic approaches.

### Synthesis of main findings

Across DMT-specific studies, DMT was most consistently associated with improvements in domains related to bodily awareness, emotional expression, and interpersonal functioning, rather than with robust or uniform reductions in core psychopathological symptoms. In psychotic disorders, particularly schizophrenia spectrum conditions, DMT-specific studies reported improvements in embodied, relational, and engagement-related outcomes, with more variable effects on core negative symptoms, when DMT was integrated into standard care (12, 14). Complementary evidence from non-DMT creative and expressive arts interventions also suggests benefits in psychosocial functioning and emotional engagement in psychotic disorders (e.g., 11). These findings are clinically relevant given the limited responsiveness of negative symptoms to pharmacological treatment. Qualitative and mixed-methods investigations further highlighted experiential benefits associated with DMT in psychotic disorders, including increased emotional expressivity, stress relief, enhanced sense of agency, and improved relational attunement (13, 16, 17). Although such outcomes are not always captured by standardized symptom scales, they may represent clinically meaningful changes in populations characterized by social withdrawal, affective flattening, and impaired interpersonal functioning. In EDs, the available evidence base was smaller and predominantly exploratory and largely derived from non-DMT creative and expressive arts interventions. These studies nevertheless reported improvements in body image, emotional regulation, alexithymia, and subjective well-being (15, 20, 21, 29). Although direct evidence for DMT in ED populations remains limited, these findings highlight the relevance of embodied and experiential processes that constitute central therapeutic targets of DMT. These findings are particularly noteworthy given that disturbances of embodied self-experience, interoceptive awareness, and emotional processing represent core psychopathological features of EDs that are often resistant to purely cognitive or nutritionally oriented interventions. Importantly, while findings in ED populations appear promising, they should be interpreted as preliminary and largely indirect, given the limited number of studies explicitly evaluating DMT as a standalone intervention in this clinical group.

The present findings are consistent with previous systematic reviews examining body-oriented and creative arts therapies in psychiatric populations. For example, Koch et al. (3) reported that dance and dance/movement therapy interventions are associated with improvements in psychological well-being and affect regulation across clinical and non-clinical populations. Similarly, Millman et al. (5, 30) highlighted the potential role of movement-based interventions in supporting emotional processing and embodied awareness in mental health contexts. Reviews of art-based and body-oriented therapies in eating disorders have also emphasized the importance of addressing disturbances in bodily self-experience and interoception through experiential interventions (29). Taken together, these findings suggest that movement-based and other creative therapeutic modalities may complement verbally mediated treatments by targeting embodied dimensions of psychopathology.

### Embodied and mechanism-oriented interpretation

From a mechanistic perspective, referring to the underlying psychological and embodied processes through which an intervention may produce therapeutic change, the potential effects of DMT can be understood within embodied and relational models of mental functioning. By directly engaging sensorimotor processes, proprioception, rhythm, and affective expression through movement, DMT provides access to pre-reflective levels of experience that are frequently disrupted in severe psychiatric conditions (5, 10, 31). In psychotic disorders, movement-based relational processes such as mirroring, synchronization, and shared rhythm may support social cognition and interpersonal engagement, potentially contributing to improvements in negative symptoms and psychosocial functioning observed in DMT-specific studies (e.g., 12), as well as in related creative and expressive arts interventions (e.g., 11). In EDs, creative and expressive arts–based interventions more broadly appear to facilitate a renegotiation of the relationship with the body as a lived and expressive entity rather than as an object of control or evaluation. Studies focusing on body-oriented and experiential outcomes suggest that movement- and art-based exploration within a therapeutic frame may enhance interoceptive awareness, reduce bodily avoidance, and support emotional integration (15, 20). Although direct mechanistic evidence for DMT in ED populations remains limited, these findings align with core theoretical assumptions of DMT and support its conceptual relevance in this clinical domain. Recent work further indicates that body-oriented and experiential creative interventions may reduce alexithymia and improve affective awareness, potentially complementing standard treatment approaches in ED populations (21).

### Intervention characteristics and clinical context

The reviewed literature highlights substantial variability in the characteristics of creative and expressive arts–based interventions, including DMT, in terms of duration, frequency, intensity, and therapeutic focus. While longer-term interventions were more commonly associated with sustained improvements, emerging evidence suggests that even brief or single-session movement- or art-based expressive interventions may yield meaningful short-term benefits, particularly in acute psychiatric settings (25, 27). These findings underscore the flexibility of DMT within the broader spectrum of creative and expressive arts therapies and its potential applicability across diverse clinical contexts, including inpatient, residential, and early intervention services. However, this heterogeneity complicates the identification of specific active components and limits comparability across studies. Inconsistent reporting of intervention structure, therapist training, and theoretical orientation further restricts reproducibility and synthesis, highlighting the need for standardized reporting frameworks in future research (32).

### Methodological considerations

The overall quality of the available evidence base on creative and expressive arts–based interventions remains limited. Although several randomized and controlled studies were identified, many investigations relied on non-randomized or quasi-experimental designs with small samples and heterogeneous and often non-comparable outcome measures (13, 20). Risk of bias was moderate to high in several studies, particularly with respect to confounding, blinding, and outcome reporting. Moreover, discrepancies between quantitative and qualitative findings were evident. While standardized measures sometimes showed modest or inconsistent effects, qualitative data frequently indicated meaningful experiential change. This divergence highlights the challenge of evaluating embodied interventions using outcome measures primarily designed for symptom-based treatments and underscores the need for more integrative, process-sensitive, and embodied assessment strategies that are better aligned with the theoretical and clinical targets of DMT and related embodied interventions.

### Limitations

#### Methodological quality and risk of bias

Several limitations of the present systematic review should be acknowledged. First, the overall methodological quality of the included studies was variable, with a predominance of small sample sizes, non-randomized designs, and heterogeneous intervention protocols. These characteristics substantially limit the strength of causal inferences regarding the effectiveness of creative and expressive arts–based interventions, including DMT. In addition, risk of bias was moderate to high in several studies, particularly among non-randomized and observational designs. Common sources of bias included potential confounding, lack of blinding, incomplete outcome reporting, and attrition. These methodological issues were especially salient in studies involving eating disorder populations, where the available evidence remains preliminary and largely exploratory.

### Heterogeneity of outcomes and interventions

Second, there was marked heterogeneity in outcome measures across studies, encompassing clinical symptom scales, embodied and somatic indicators, psychosocial functioning, and qualitative experiential outcomes. While this diversity reflects the multidimensional nature of creative and expressive arts–based interventions, it limited the feasibility of quantitative synthesis across most outcome domains. In particular, outcomes related to embodied experience and therapeutic processes were frequently assessed using non-standardized or study-specific instruments, complicating cross-study comparison and synthesis. Furthermore, heterogeneity in the interventions themselves represents an additional limitation. Included studies varied widely in terms of intervention duration, session frequency, therapeutic focus, and format (group versus individual). Detailed descriptions of intervention components, theoretical orientation, and therapist training were not consistently reported, reducing reproducibility and limiting the identification of specific active components underlying observed effects.

### Search strategy and distribution of evidence

Third, the search was restricted to PubMed/MEDLINE. Although this database provides broad coverage of biomedical and psychiatric research, the restriction to a single database may have increased the risk of missing relevant publications. This limitation was partially mitigated by manual screening of reference lists from all included studies and relevant reviews. Finally, although the review aimed to include a range of psychiatric populations, the distribution of evidence across diagnostic groups was uneven. The literature on psychotic disorders was more extensive than that on EDs, limiting the generalizability of conclusions across conditions and highlighting the need for more focused and adequately powered research in underrepresented populations. Taken together, these limitations indicate that the findings of the present review should be interpreted primarily as descriptive and hypothesis-generating rather than confirmatory.

### Clinical and research implications

The findings of this review suggest that creative and expressive arts–based interventions may represent a valuable adjunctive component within multidisciplinary mental health care. Within this broader framework, DMT appears particularly relevant for individuals experiencing difficulties with verbal expression, emotional awareness, or bodily integration. The available evidence suggests potential relevance of DMT in both psychotic disorders and EDs, although conclusions regarding efficacy must remain cautious. Future research should specifically prioritize well-powered randomized controlled trials explicitly designed to evaluate DMT in eating disorder populations, in order to determine whether the promising embodied and affective effects observed in broader expressive and body-oriented interventions can be attributed to DMTspecific mechanisms. Particular attention to ED populations is warranted, given the preliminary but promising findings identified in this review and the current imbalance in the distribution of evidence across diagnostic groups.

### DMT within the broader field of creative arts therapies

Within the broader field of creative and expressive arts therapies, DMT occupies a distinctive position due to its direct engagement with bodily processes and sensorimotor experience. While other modalities such as art therapy or music therapy rely primarily on symbolic or auditory channels of expression, DMT explicitly targets embodied awareness, movement patterns, and interpersonal attunement.

## Conclusions

This systematic review examined the current evidence on creative and expressive arts–based interventions in psychiatric populations, with particular attention to DMT and a focus on psychotic disorders and EDs. Overall, the available literature suggests that creative and expressive arts–based interventions may be associated with potentially beneficial effects on embodied experience, emotional expression, and psychosocial functioning, domains that are often insufficiently addressed by standard psychiatric treatments. Within this broader field, DMT-specific studies appear particularly relevant for outcomes related to bodily awareness, affective expression, and interpersonal engagement. In psychotic disorders, DMT appears to hold promise for improving negative symptoms, social engagement, and bodily awareness. In EDs, the available evidence, largely derived from non-DMT creative and expressive arts interventions, suggests potential benefits in body image, emotional regulation, and affective awareness, highlighting the relevance of embodied and experiential processes that constitute key theoretical targets of DMT. These findings are clinically relevant given the central role of embodied and relational disturbances across these conditions, which are often insufficiently addressed by standard treatment approaches. However, conclusions regarding efficacy must remain cautious due to substantial methodological heterogeneity and variable study quality. The current evidence base is characterized by small sample sizes, diverse intervention protocols, and inconsistent outcome measures, underscoring the exploratory nature of the field. Future research should prioritize well-designed randomized controlled trials, standardized reporting of DMT intervention characteristics, and the inclusion of outcome measures capable of capturing embodied, interoceptive, and relational processes. Particular attention is warranted for ED populations, which remain underrepresented despite preliminary and promising findings. In conclusion, while the current evidence does not yet support definitive efficacy claims, DMT may represent a valuable and flexible adjunctive intervention within multidisciplinary mental health care, particularly when integrated with standard treatments and tailored to embodied and relational therapeutic targets. In EDs, conclusions regarding DMT remain tentative and should be understood primarily as hypothesis-generating rather than confirmatory, due to the limited availability of DMT-specific controlled studies. Continued methodological refinement and mechanism-informed research will be essential to clarify its role and optimize its clinical application across psychiatric settings.

## Data Availability

The original contributions presented in the study are included in the article/supplementary material. Further inquiries can be directed to the corresponding author.
